# Do lower urinary tract symptoms predict cardiovascular diseases in older men? A systematic review and meta-analysis

**DOI:** 10.1007/s00345-015-1560-1

**Published:** 2015-05-14

**Authors:** Iris I. Bouwman, Maarten J. H. Voskamp, Boudewijn J. Kollen, Rien J. M. Nijman, Wouter K. van der Heide, Marco H. Blanker

**Affiliations:** Department of General Practice, University Medical Center Groningen, Antonius Deusinglaan 1, 9713 AV Groningen, The Netherlands; Department of Urology, University Medical Center Groningen, Groningen, The Netherlands

**Keywords:** Ageing male, Cardiovascular diseases, Lower urinary tract symptoms, Systematic review/meta-analysis

## Abstract

**Purpose:**

To study the incidence of CVD in men at risk, with and without LUTS.

**Methods:**

We searched all longitudinal studies describing the association between LUTS and CVD (mortality) in October 2013 and December 2014 using MEDLINE, EMBASE, and the Cochrane Library Central Register. PRISMA criteria were met.

**Results:**

We included five studies with 6027 men with LUTS and 18,993 men without LUTS in the meta-analyses, with a follow-up period varying from 5 to 17 years. Studies totalled 2780 CVD events. No clear association between CVD and LUTS was demonstrated [pooled effect size: hazard ratio 1.09 (95 % CI 0.90–1.31); *p* = 0.40]. Two other studies reported the association between nocturia and (CVD) mortality. CVD-specific mortality risk was approximately two times higher for Japanese men with nocturia (357 men aged 70 years and over, 5-year follow-up). A univariable association between nocturia and all-cause mortality was found in Dutch men, but not in age-adjusted analyses (1114 men aged 50–78 years, 13-year follow-up).

**Conclusion:**

This meta-analysis conducted on longitudinal studies does not confirm LUTS to be a predictor of CVD in men without a history of CVD, despite the observed association between LUTS and CVD in cross-sectional studies.

## Introduction

Cardiovascular diseases (CVD) are the leading cause of death globally and cause high morbidity [1]. The increasing prevalence of CVD in the ageing population necessitates the timely identification of people who are at risk [2]. It has been suggested that lower urinary tract symptoms (LUTS) are associated with CVD and may predict cardiovascular events. These conditions share multiple risk factors such as obesity, diabetes, hypertension, smoking, and advanced age [3–7]. If so, therapeutic interventions to improve LUTS at an early stage may be considered to prevent morbidity and mortality due to CVD. Also screening of men with LUTS on cardiovascular diseases will be meaningful.

The pathogenesis of LUTS is considered to be multifactorial, in which age-related changes in prostate, bladder structure, and bladder function seem to play a central role. Vascular diseases such as atherosclerosis and endothelial dysfunction in the pelvic vascular system might contribute to bladder dysfunction with age [3]. The risk factors for vascular diseases and atherosclerosis might also have an impact on LUTS via other mechanisms [8]. For instance, increased sympathetic activity and/or a1-adrenoreceptor activity is suggested as the common pathway for both hypertension and LUTS [9]. Nicotine, but also waking by nocturia [10, 11], increases the sympathetic nervous system activity and may contribute to LUTS via an increase in the tone of the prostate [12, 13]. Hypertension and heart failure can cause fluid shifts and hormonal and autonomic nervous disturbances, causing LUTS. Finally, neurogenic bladder dysfunction with detrusor underactivity can cause LUTS in patients with diabetes mellitus [3].

Previous studies already reported on the association between LUTS and CVD in cross-sectional settings, performed in both clinical and community-based populations [3–7, 9, 14, 15]. It remained unclear whether this association between LUTS and CVD reflects a true increased risk of CVD, or if it is mainly explained by, for example, age, which is strongly associated with both LUTS and CVD [1, 16]. The outcomes are heterogeneous [3–7]. More recently, longitudinal studies have been published.

To better understand the possible relationship between LUTS and CVD and to know whether LUTS is a precursor for CVD in people without a history of CVD, we performed a systematic review and meta-analysis based on longitudinal studies. We were especially interested in determining whether LUTS could be considered as a predictor of CVD.

## Methods

### Search strategy/study selection

We conducted a systematic review of the literature. Potentially relevant studies were identified through a structured literature search of MEDLINE, EMBASE, Cochrane Database of Systematic Reviews, and Cochrane Central Register of Controlled Trials using Medical Subject Headings (MeSH) and free-text keywords. The terms LUTS AND CVD AND (cohort studies OR longitudinal studies) were combined, using various synonyms presented in “Appendix 1”.

We identified articles eligible for further review by performing an initial screen of identified titles and abstracts, followed by a full-text review. In addition, we searched the reference list of all identified relevant publications on October 20, 2013, and repeated the complete search on December 10, 2014.

### Inclusion, exclusion, and quality score criteria

Two investigators (IB and MV) independently assessed literature eligibility. Articles were considered for inclusion if: (1) the study included original data, published in a peer-reviewed journal (i.e. not review articles, or meeting abstracts); (2) the study was a cohort study (prospective cohort or historical cohort) consisting of male human adults; and (3) the authors reported the risk estimates of cardiovascular morbidity in LUTS patients compared with non-LUTS patients. Selected studies included all types of CVD, cardiovascular risk, and LUTS.

To assess the methodological quality of included studies, a structured form was used. We chose to apply a score based on a criterion list which has been previously used in systematic reviews of observational data [17, 18]. We tailored the list by adding criteria on the completeness of data on LUTS and CVD. The list included six items on external validity, five items on internal validity, and seven items on informativity (“Appendix 2”). IB and MV independently scored all items either positive (scored as 1) or negative (scored as 0).

### Data extraction

Two reviewers (IB and MV) independently extracted the following information about the studies: study characteristics (study name, authors, publication year, journal, study site, follow-up years, and number of participants), participants’ characteristics (mean age or age range), main exposure LUTS (IPSS, AUA, and nocturia), main outcome cardiovascular disease/risk (morbidity, risk, types, assessed by self-report, and medical records), and analysis strategy (statistical models, covariates included in the models). Disagreement about data extraction or the quality score of the included studies was resolved by consensus. When no or insufficient information was provided in the article, earlier publications on the same study were used for collecting lacking information, if available. For pooling of data, on our request, we received additional information from two studies (personal communication—Wehrberger et al. [19] and Lin [20]). This concerned information about hazard ratios for CVD risk in men with moderate to severe LUTS, compared to men with no or mild LUTS.

### Meta-analyses

We selected studies that compared the incidence of a CVD between individuals with and without LUTS. Studies including patients with only (cardiovascular) mortality as an endpoint were considered not fully representative of CVD. Therefore, we did not include these studies in the meta-analysis but analysed these studies separately.

In all analyses, we considered methodological homogeneity. If data were pooled, statistical heterogeneity was assessed using the *I*^2^ index. In case of substantial heterogeneity (*I*^2^ ≥ 50 %), a random effects model was applied. A fixed effects model was used when *I*^2^ < 50 %. Weighted hazard ratios (HR) were presented with 95 % confidence intervals (95 % CI). Statistical analysis was performed with RevMan 5.3.

## Results

### Selection of studies

**Fig. 1:**
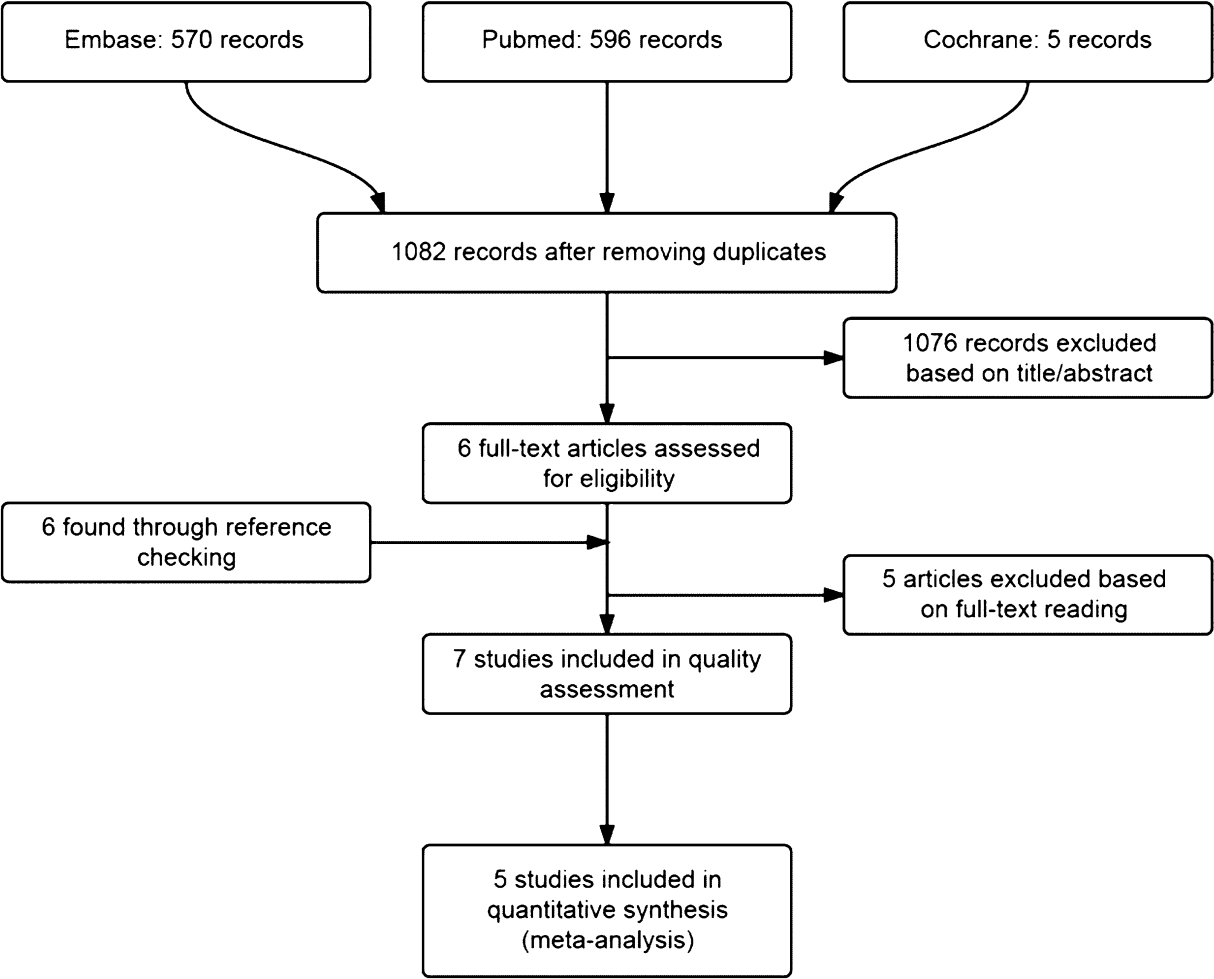
Flowchart of study selection

We reviewed seven studies (with 25,982 male participants) selected from 1082 initially identified publications (Fig. 1). This selection included four community-based studies [10, 19–21] and one primary care study [22]. In two other studies (1471 participants), nocturia and (CVD) mortality were assessed [23, 24]. Background information for these studies is presented in Tables 1 and 2.

**Table 1:** General study characteristics

Ref. no.	Year	Country	Participants	Age	Origin	Inclusion criteria	Exclusion criteria	Responders/total population *N*/*N* %	Study period	Follow-up time (mean*/median^~^)
[19]	2011	Austria	Men	Mean 47.8 (SD 11.5)	CB	Employees of large companies undergoing free of charge in. The city of Vienna. Men >30 years	Men with a history coronary heart disease, cardiac failure, intermittent claudication, atrial fibrillation, left ventricular hypertrophy	2092/?	2001–2008	6.1 years* (SD 1.5)
[10]	2012	USA	Men	Median 51.88; (Q1–Q3 44.8–62.8)	CB	Random sample of men aged 40–79 years in 1990, Olmsted County, MN, USA	Previous history of PCa, prostatectomy, or bladder cancer, bladder disorders or surgery, and urethral disorders or surgery	2447/3874 (63 %)	1990–2007	17.1 years^~^ (25th–75th: 15.2–17.4)
[20]	2013	Taiwan	Men and women	Mean 47 (SD 15)	CB	Nationwide health insurance enrolees, subjects with codes of LUTS in service claims	Subject with CVD codes	27,318/?	2001–2009	6.6 years* (SD 1.5)
[22]	2014	Netherlands	Men	Mean 56 (SD 12)	PCP	Male population aged >50, in a primary care population in the Netherlands	PCa, history of CVD	6615/?	1998–2008	8.2 years*, 11.0^~^ (25th–75th: 5.2–11.0)
[23]	2011	Netherlands	Men	Median 60.8 (Q1–Q3 56.0–66.2)	CB	All men aged 50–78 years from Krimpen aan den IJssel, The Netherlands	History of TURP, prostatectomy, PCa or bladder cancer, neurogenic bladder disease, or negative advice from the GP based on poor health	1114/3924 (28.4 %)	1995–2010	13.4 years^~^ (25th–75th: 10.3–14.1)
[21]	2014	Netherlands	Men	Mean 61.4 (SD 6.6)	CB	All men aged 50–78 years from Krimpen aan den IJssel, The Netherlands	History of prostatectomy, prostate or bladder cancer, neurogenic bladder disease, or who were unable to complete questionnaires or visit the healthcare centre	1610/3924 (41 %)	1995–2003	6.35* (range 0.1–8.34 years) total: 7945 person-years
[24]	2010	Japan	Men and women	Mean 76 (SD 4.6)	CB	All men aged >70 from Tsurugaya, Japan	Not joining the NHI system	784/2925 (26.8 %)	2003–2008	5 years*

*CB* community based, *PCP* primary care population, *CVD* cardiovascular disease, *GP* general practitioner, *PCa* prostate carcinoma, *NHI* National Health Institute

**Table 2:** Characteristics of studies: measurement instruments, applied definitions, and confounders used in the adjusted analysis

Ref. no.	LUTS	Nocturia	CVD	Mortality	Confounders
[19]	IPSS categories	–	ICD codes: I20–25, I60–65	–	Age, sex, DM, cholesterol, BMI, SBP, DBP, uric acid
[10]	–	NVF ≥ 2	Medical records: sudden cardiac death, MI and angiographically diagnosed coronary disease	Death certificates, autopsy reports, obituary notices, electronic death certificate data from the state of Minnesota	Age, DM, BMI, α-blockers, 5α-reductase inhibitors, OAB medications, HT
[20]	ICD-9-CM codes: 596.51, 788.4, 625.6, 788.32, 788.31, 788.36, 788.43, 788.33, 788.2, 788.6, 788.35, 600	–	ICD-9 codes: 410, 411, 430, 431, 433–436	Subjects who withdrew the NHI enrolment within 30 days after discharge from the last hospitalization were presumed dead, and the discharge date was designated as the date of death	Hypertension, diabetes, hyperlipidaemia, and age
[22]	ICPC codes: U02, U05, U07, U13, U29, Y06, Y85	–	ICPC codes: K74, K75, K76, K89, K90	Medical records from the Registration Network Groningen	HT, DM, obesity, alcohol, dyslipidaemia, medication: TCA, antipsychotics, antihypertensives, diuretics, statins, LUTS medication.
[23]	–	IPSS NVF ≥ 2	–	Patient files in the GP database	Age, alcohol, tobacco, DM, albuminuria, obesity, hypertension, COPD, cardiac symptoms,
[21]	IPSS categories	–	ATC definitions for AMI, stroke, sudden death	Patient files in the GP database	Age, alcohol, tobacco, DM, obesity, hypertension, ED
[24]	–	NVF ≥ 2	Questionnaire	NHI claims history files	Age, sex, alcohol, tobacco, DM, medication, disease history, CVD history, nephropathy, malignant disease, BMI, tranquilizer, hypnotics, diuretics, functional reach

*IPSS* International Prostate Symptom Score, *ICD* International Classification of Diseases, *NVF* nocturnal voiding frequency, *NHI* National Health Insurance, *CHD* cardiac heart disease, *CF* cardiac failure, *CI* intermittent claudication, *DM* diabetes mellitus, *BMI* body mass index, *SBP* systolic blood pressure, *DBP* diastolic blood pressure, *OAB* overactive bladder, *HT* hypertension, *(A)MI* myocardial infarct, *GP* general practitioner, *COPD* chronic obstructive pulmonary disease, *ICPC* International Classification of Primary Care, *U02* frequency, *U05* other voiding symptoms, *U07* other urine symptoms, *U13* other bladder symptoms, *U29* other urine tract symptoms, *Y06* prostate symptoms, *Y85* benign prostate hypertrophy, *K74* angina pectoris, *K75* acute myocardial infarction, *K76* other/chronic coronary heart disease, *K89* transient cerebral ischaemia, *K90* cerebrovascular accident, *ED* erectile dysfunction, *ATC* Antithrombotic Trialists’ Collaboration

### Methodological quality assessment and description of selected studies

Table 3 shows the results of the quality assessment. The proportions scored positive were 0.71 on external validity, 0.74 on internal validity, and 0.88 on informativity. In 89 % of the items, there was a positive agreement, whereas in 11 % of the items consensus was reached after discussion between the two reviewers. None of the studies scored positive on all validity criteria.

**Table 3:** Quality score criteria

Ref. no.	Year	External validity							Internal validity						Informativity								
		a	b	c	d	e	f	Sum	g	h	i	j	k	Sum	l	m	n	o	p	q	r	Sum	Disagreement
[19]	2011	+	+	−	−	−	+	3	−	+	+	+	+	4	+	+	+	+	+	+	−	6	c, i, r
[10]	2011	+	+	−	−	−	+	3	−	+	+	+	+	4	+	+	+	+	+	+	+	7	i, o, r
[20]	2013	+	+	+	+	+	+	6	−	+	+	+	+	4	+	+	+	+	+	+	+	7	d, h
[22]	2014	+	+	−	−	+	+	4	−	+	+	+	+	4	+	+	+	+	+	+	+	7	h
[23]	2012	+	+	+	+	+	+	6	−	+	+	+	+	4	+	+	+	+	−	+	+	6	c, e
[21]	2014	+	+	+	+	+	+	6	−	+	+	+	+	4	+	+	+	+	+	+	+	6	r
[24]	2010	+	−	−	−	−	+	2	−	+	−	−	+	2	+	+	+	−	−	+	−	4	j, o
Total		6	5	2	2	3	6		0	6	5	5	6		6	6	6	5	4	6	4		
Mean								4.3						3.7								6.1	

Items a–r refer to the quality score criteria listed in “Appendix 2”

### LUTS and CVD

The five studies included in the meta-analysis compared CVD outcomes between in total 6027 men with LUTS and 18,993 men without LUTS. Two studies reported a median follow-up of 17.1 and 11.0 years [10, 22]; three other studies reported a mean duration of follow-up of 6.1, 6.35, and 6.6 years [19–21]. During this follow-up, a total of 2780 CVD events occurred: 646 in men with LUTS (17 %), compared to 1775 in men without LUTS (10 %). Lightner et al. [10] only report a total of 359 men with CVD in the follow-up group.

**Fig. 2:**
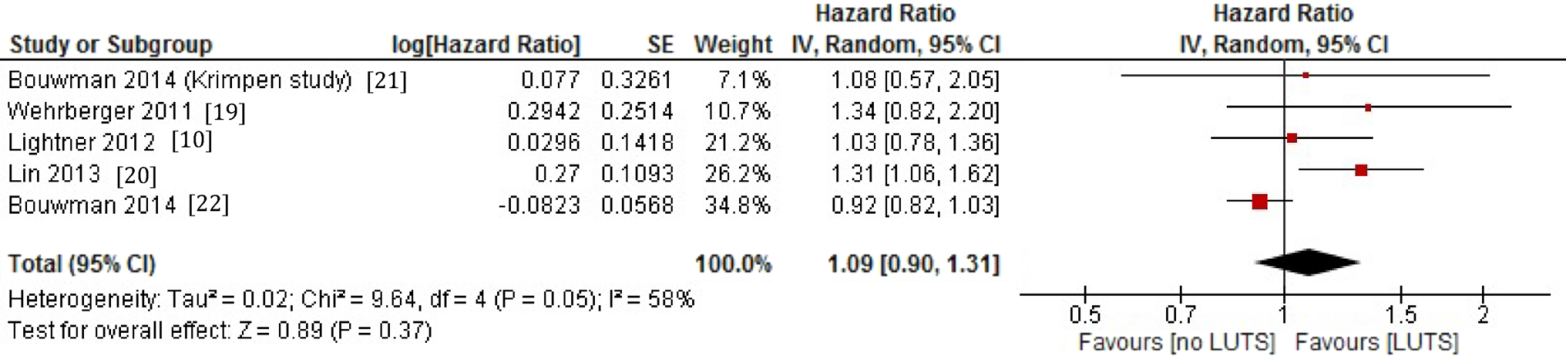
Risk (hazard ratio) of CVD according to LUTS status. Pooled hazard ratio with 95 % CI resulting from meta-analyses using generic inverse variance random effects model. Presentation in order of publication year

Figure 2 shows the results of the pooled analyses with an estimated hazard ratio of 1.09 (95 % CI 0.90–1.31), *p* = 0.40.

### Nocturia and (cardiovascular) mortality

Two studies described the CVD mortality risk for men with and without nocturia [23, 24]. Nakagawa et al. [24] conducted a community-based observational study and followed 784 men aged 70 years and older, of whom 359 had nocturia. During a 5-year observation period, these men had a two times greater risk to die from CVD than the men without nocturia: HR 1.98, 95 % CI 1.09–3.59, *p* = 0.03. In this analysis, the authors adjusted for sex, alcohol, tobacco, diabetes, medication, CVD history, nephropathy, malignant disease, BMI, and functional reach.

Van Doorn et al. [23] conducted a similar observational study with 1114 men aged 50 years and older with an extended follow-up period of 13.4 years. At baseline, 731 had no nocturia and 383 had nocturia. During a median follow-up period, there was an association between nocturia and increased mortality rate in the univariable analysis (HR 1.63, 95 % CI 1.20–2.21, *p *= 0.002). This association between nocturia and all-cause mortality was not found in the model adjusting for age, COPD, hypertension, and smoking: the adjusted HR was 1.03 (95 % CI 0.75–1.42).

## Discussion

This systematic review and meta-analysis of longitudinal studies does not confirm that LUTS is a precursor for CVD, as has been suggested from cross-sectional studies [3–7, 9, 14, 15]. This might implicate that the presence of LUTS in older men is no reason to start preventive cardiovascular treatment. At least, raising public awareness on CVD in men with LUTS seems not to have a scientific ground.

The number of available studies on this topic was limited; only seven cohort studies with 25,982 participants between LUTS and CVD, or nocturia and (cardiovascular) mortality were available for review. In general, longitudinal studies are more laborious and expensive to perform, but vital to analyse possible causal relationships. Earlier cross-sectional studies showed apparently clear association between LUTS and cardiovascular risk factors [3, 4, 6, 7, 9, 14, 15]. No inference can be made on the causality of such associations.

The results of longitudinal studies should also be considered with some caution. Especially, the applied statistical methods need to be sound. The preferred statistical method is survival analyses. In such analyses, time to event is the outcome. This is especially important for severe outcomes, such as CVD. In a very recent longitudinal study on this topic [7], authors concluded that LUTS severity predicts CVD. In that study, however, authors applied logistic regression analyses, instead of survival analyses. We believe that this statistical method is inferior to survival analyses and may have led to an overestimation of the studied association. Comparing proportions of events in study groups using odds ratios/logistic regression ignores time as an important factor [25]. Moreover, due to this difference, that study could not be included in the current meta-analysis.

Longitudinal studies may provide a temporal sequence of events required for revealing time-dependent relationships between LUTS and CVD or mortality. In this respect, we focussed on this temporal sequence and not on the reverse sequence of the possible negative impact of CVD (treatment) on the development of LUTS.

The absence of a clear association described in this review needs to be interpreted with some caution. The studies included in this review were based on general population samples, health registries, and one general practice population. In such populations, men with severe LUTS, as well as high-risk patients, may be underrepresented. For example, in the Wehrberger study, only 1 % of the participants had severe LUTS. This small group had a higher risk of CVD than men without LUTS [19]. Adding this group to the men with moderate LUTS, for the purpose of pooling the data in the current meta-analysis, revealed no significant difference compared to men without LUTS. Longitudinal studies in high-risk patients are lacking. Surprisingly, no data from secondary or tertiary care settings were found.

Second annotation is made on the applied definitions of both LUTS and CVD, which differed between studies. As defined by the European Association of Urology (EAU), LUTS is a very broad concept which incorporates a range of micturition symptoms [26]. Likewise, CVD includes a broad number of diseases, not always sharing the same aetiology. Included studies did not always specify for myocardial infarction, peripheral vascular disease, or stroke. Being unable to find an association in our review could also be due to these different aetiologies between and within LUTS and CVD. On the other hand, from a methodological point of view, applying different definitions could enhance making firm conclusions, if a clear association would have been shown in all studies. In the current review, in only one out of five studies, a significant association was found [20].

Finally, as both LUTS and CVD are believed to have a multifactorial origin [1, 16], other possible causes of CVD, such as nicotine abuse, hyperlipidaemia, BMI, and alcohol abuse, should also be considered in studying the association between LUTS and clinical CVD. Although all studies reported implementing adjustments for confounding factors such as age, gender, and smoking status, not all known cardiovascular risk factors were included in the longitudinal analyses. This might have had impact on the described associations.

So, although our review suggests that LUTS do not predict CVD in men without a history of CVD, the association between LUTS and CVD could not be ruled out.

Next to these general concerns on the available data, some methodological issues need to be mentioned as well. We have searched relevant databases, but with our search strategy, individual studies might have been overlooked. For that reason, we also checked the reference lists. We have applied a criteria list for the methodological quality assessment, previously used in comparable reviews [17, 18]. This included some arbitrary items, for example, on the availability of follow-up percentages and information on losses to follow-up. It is impossible to provide such data, for example, from registry studies, resulting in a lower quality score for such studies. Next, for the meta-analyses, we have chosen to perform pooled analyses, despite the clinical heterogeneity present. Evaluating the separate studies, however, would have led to the same conclusions, as four of the five studies showed no association. Due to the statistical heterogeneity, we needed to adjust the analyses, by performing a random effects model. We could not perform subgroup analyses.

## Conclusion

Despite the limitations addressed, we conclude that the presence of LUTS does not predict CVD in older men without a history of CVD. Raising awareness for CVD in men with LUTS in the general population seems to have no evidence base. No information was found on this association in more selective clinical samples. To further address LUTS as a possible marker of underlying CVD, high-quality prospective research in a clinical setting is needed. This should be done applying generally accepted definitions of LUTS and CVD.

## Appendix 1

**Table:** Search terms used

Keyword	Synonym
CVD	(“Cardiovascular Diseases” [Mesh:NoExp]) OR (“Heart Diseases” [Mesh:NoExp]) OR (“Arrhythmias, Cardiac” [Mesh]) OR (“Heart Arrest” [Mesh]) OR (“Heart Failure” [Mesh]) OR (“Myocardial Ischemia” [Mesh]) OR (“Pulmonary Heart Disease” [Mesh]) OR (“Vascular Diseases” [Mesh:NoExp]) OR (“Arteriosclerosis” [Mesh]) OR (“Carotid Stenosis” [Mesh]) OR (“Cerebrovascular Disorders” [Mesh]) OR (“Embolism and Thrombosis” [Mesh]) OR (“Hypertension” [Mesh]) OR (“Myocardial Ischemia” [Mesh])OR “cardiovascular disease” OR “coronary heart disease” OR “ischemic heart disease” OR “coronary artery disease” OR “hypertensive heart disease” OR “cor pulmonale” OR “cerebrovascular disease” OR “peripheral arterial disease” OR “stroke” OR “atherosclerosis” OR “myocardial ischemia” OR “acute coronary syndrome” OR “angina pectoris” OR “coronary disease” OR “coronary artery disease” OR “coronary occlusion” OR “coronary stenosis” OR “coronary thrombosis” OR “myocardial infarction” OR “sudden death”
LUTS	(“prostatic Hyperplasia” [Mesh]) OR (“Lower Urinary Tract Symptoms” [Mesh]) OR (“Urination Disorders” [Mesh]) OR (”Urinary bladder neck obstruction” [MeSH])OR (“Urinary Bladder, overactive” [Mesh])OR (“Polyuria” [Mesh]) OR “dysuria” OR “nocturia” OR “prostatism” OR “overactive urinary bladder” OR “overactive bladder” OR “urinary incontinence” OR “urgency” OR “Hesitancy” OR “lower urinary tract symptoms” OR “benign prostatic hyperplasia” OR “benign prostatic hypertrophy” OR “prostatic hyperplasia” OR “prostatic hypertrophy” OR “bladder outlet obstruction” OR “voiding dysfunction” OR “urinary bladder neck obstruction” OR “prostatic Hyperplasia” [Mesh] OR “Lower Urinary Tract Symptoms” [Mesh] OR “Urination Disorders” [Mesh] OR “Urinary bladder neck obstruction” [MeSH] “Urinary Bladder, overactive” [Mesh] “Polyuria” [Mesh] OR “dysuria” OR “nocturia” OR “prostatism” OR “overactive urinary bladder” OR “overactive bladder” OR “urinary incontinence” OR “urgency” OR “Hesitancy” OR “lower urinary tract symptoms” OR “benign prostatic hyperplasia” OR “benign prostatic hypertrophy” OR “prostatic hyperplasia” OR “prostatic hypertrophy” OR “bladder outlet obstruction” OR “voiding dysfunction” OR “urinary bladder neck obstruction”
Longitudinal studies	“Longitudinal Studies” [Mesh] OR “Longitudinal Studies”
Cohort studies	“Cohort Studies” [Mesh] OR “Cohort Studies”

## Appendix 2

**Table:** Quality score criteria and informativity^a^

*External validity*	
Selection of the study population	
A	Clear description of the research population?^b^
B	Inclusion and exclusion criteria described?^c^
Participants and non-responders	
C	Response rate >70 % or sufficient information on non-responders?^d^
D	Is there sufficient information about the follow-up percentages and comparison of who were and were not lost to follow-up?
Relationship with source population?	
E	Extrapolating results possible for the complete population?^e^
Description of the study period	
F	Clear description of the study period?
*Internal validity*	
Data collection	
G	Data prospectively collected?
Measurement instrument	
H	Measuring instrument for LUTS^f^
I	Measuring instrument for CVD^g^
J	Are definitions of the diseases stated?
Confounders	
K	Confounders described?^h^
*Informativity* ^i^	
L	Clear theoretical introduction with relevant references to support the research question?
M	Aims of the study clearly described?
N	Research questions being answered?
O	Definition of LUTS clearly described
P	Definition of CVD clearly described
Q	Clear description of the way data were analysed?
R	Enough original data to evaluate their interpretation

^a^Items were scored positive if clear information was presented in the articles. Unclear data are presented as “?” and consequently scored negative for the quality score summation

^b^Clear description of source and two or more of the following: age distribution, relevant comorbidity, medication

^c^Scored positive if both inclusion and exclusion criteria were provided

^d^Sufficient information on non-responders: were reasons for non-response studied and presented, including information on age distribution, gender, main topic under study?

^e^Did the study selection procedure result in a representative sample of the study population?

^f^Data on LUTS collected through IPSS or validated questionnaire

^g^Data on CVD collected through validated instrument

^h^Description of confounders not necessarily including actual statistical adjustment for confounders

^i^Informativity was not included in the quality score
